# Impact of Drought on Cereals Infected with *Zymoseptoria tritici*, the Causal Agent of Leaf Spot Disease of Wheat: An Overview

**DOI:** 10.3390/pathogens15070741

**Published:** 2026-07-15

**Authors:** Nevzat Kılınç, Murat Dikilitaş, Canan Can, Avinash Mishra

**Affiliations:** 1 Department of Plant Protection, Faculty of Agriculture, Harran University, 63200 Şanlıurfa, Türkiye; m.dikilitas@gmail.com; 2 Department of Biology, Faculty of Arts and Sciences, Gaziantep University, 27310 Gaziantep, Türkiye; can@gantep.edu.tr; 3 CSIR—Central Salt and Marine Chemicals Research Institute, Bhavnagar 364002, India; avinash@csmcri.res.in

**Keywords:** plant pathogen, abiotic stress, metabolites, melanin, DNA damage, combined stress

## Abstract

*Zymoseptoria tritici* (Desm. Quaedvlieg & Crous), known as the wheat leaf spot disease agent, is a highly virulent fungus that induces blotch and necrosis on leaves. Although it is known to cause severe infections under humid conditions, recent observations suggest that it can also infect wheat leaves under drought and high-temperature conditions, possibly influenced by global warming. Recent findings showed that *Z. tritici* could easily tolerate various abiotic stresses, including drought, water stress, salinity, and temperature. It has been evident that the fungus can tolerate pesticide stress, as indicated by the increased frequency and number of pesticide applications throughout the growing season. Under stress conditions, the fungi, unlike crop plants, could easily tolerate stress by rapidly modifying gene expression and reducing spore production and mycelial growth without downregulating major biochemical components that play significant roles in pathogenicity and virulence. *Z. tritici* can accumulate melanin under stress conditions; therefore, an increase in pathogenicity under drought or salinity stress is not unexpected. Recent studies have shown that the pathogenicity of the fungus is increasing, and more virulent, toxin-producing pathogens might emerge in the future. Since drought and high-temperature stresses significantly affect crop plants, the adaptation of pathogenic microorganisms to these conditions could be inevitable if abiotic stress persists. Under these circumstances, the crop loss would be more pronounced. A critical aspect of this process is the assessment of DNA integrity in both wheat and the pathogen under drought stress conditions. The organism that better maintains DNA integrity is considered to exhibit greater drought tolerance. Therefore, our main target should be DNA health when developing or breeding new wheat varieties, considering double- or even multiple-stress conditions. We should finally state that we are very optimistic about generating highly stress-tolerant wheat varieties via metabolomic and proteomic approaches without compromising quality. However, the impact and combination of stress factors are becoming increasingly complex.

## 1. Introduction

Plants always face stressors throughout their life cycles. Running away from stress or keeping it at a distance is impossible because of their sessile nature. However, when exposed to stress, they use effective coping strategies, compared with those of animals and humans. Stress factors, however, do not come individually as in previous decades; they come in pairs or multiples, either sequentially or simultaneously. In each case, the defence mechanism of crop plants varies. Thus, managing stress combinations for crop plants is becoming increasingly difficult and complex as stress intensity and duration increase. The developmental stage of crop plants and interactions among stress factors may also affect crop responses. For example, interactions between abiotic and biotic stressors can be antagonistic, additive, or synergistic, and these stressors can occur simultaneously or sequentially. The possible interaction mechanisms, particularly drought, were elucidated in the following sections and in our previous work [1]. We observed that drought stress and pathogen occurrence could exert an additive effect; both stressors could individually induce their own symptoms, or crop plants could tolerate drought stress while showing resistance to pathogen stress to the best of their ability [2]. We could also observe that one stress factor triggers the other and that the combined stress exhibits a synergistic effect [3]. One stressor may suppress the effects of another, resulting in only the dominant response being observed; thus, the observed outcome may reflect either antagonistic interactions between stress factors or the overriding negative effect of a single stressor [4]. Usually, both stressors negatively affect crop plant growth and development. Pathogenic or drought stress might have a priming effect, and crop plants might develop better due to hormonal effects, as observed under virus and drought conditions [1,4,5]. Bonnet et al. [6], on the other hand, reported that the second stress may dominate plants’ transcriptional response. *Brassica nigra* (L.) W.D.J.Koch pretreatment with aphids (*Brevicoryne brassicae*) and the bacterial phytopathogen *Xanthomonas campestris* pv. *raphani* (*Xcr*) showed that the second stress dominated the transcriptional signature. However, we need to release the entire plant genome transcriptome to identify key genes for each stress factor and the combined stress factor. Sinha et al. made a similar statement that the outcome of combined stress depended on which stress factors occurred first in the plant [7]. Pandey et al. [8] reported that when abiotic and biotic stresses induce the same physiological changes, the combined effect is additive, resulting in greater damage to plants. If two stresses lead to a completely unique response, this probably means that plants use a shared pathway to save energy. Therefore, the adaptation mechanism to combined stress consisted of both shared and unique responses. The identification of genes involved in shared and unique responses under combined stress would be an important step toward developing tolerant and resistant plants.

**Figure 1:**
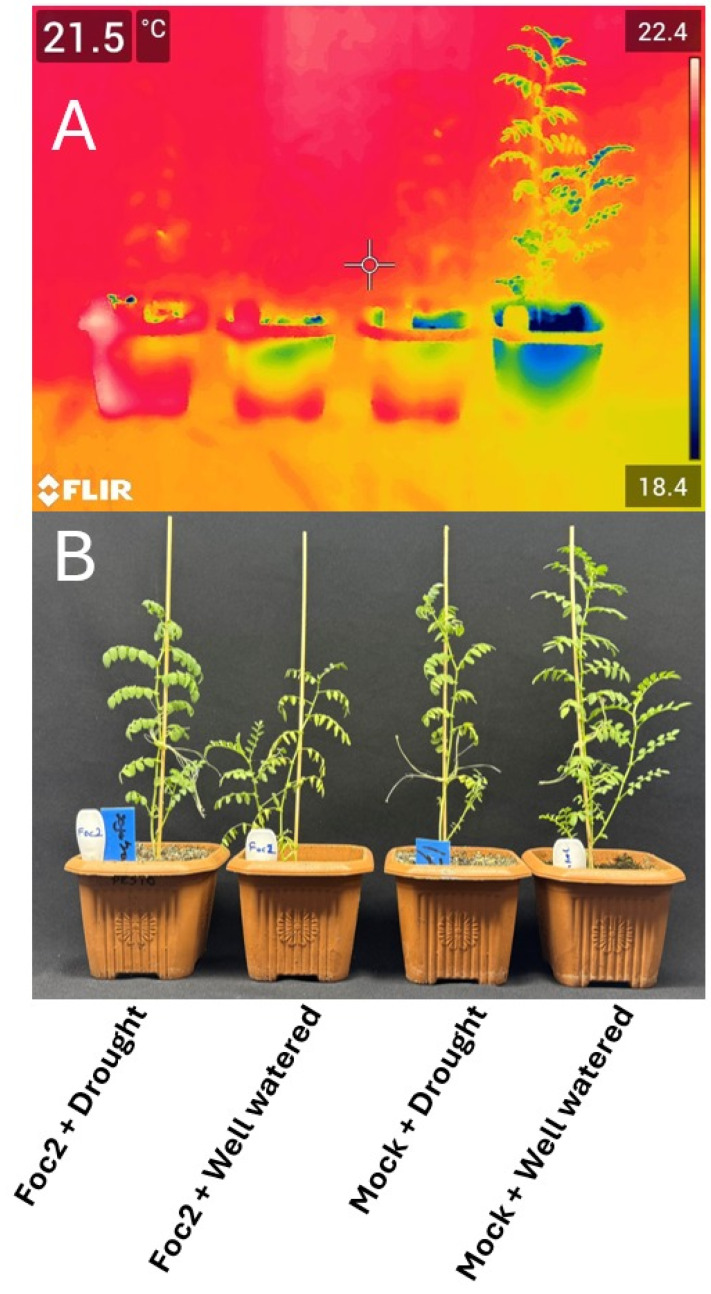
Responses of chickpea plants inoculated with *Fusarium oxysporum* f.sp. *ciceris* (*Foc*) under water stress conditions. (**A**) thermal camera results of the experimental setup; (**B**) daylight camera results of the experimental setup. Please note that the effects of the combined stress of drought and pathogens on chickpea plants are not very different from those of other plants under drought or pathogen stress conditions. However, real-time thermal imaging showed that the plants subjected to the combination of water stress and pathogen treatment emitted red light, indicating greater calorie expenditure and thus increased energy consumption under combined stress conditions. The plants under combined stress died quickly, within a few days of this picture being taken.

We have recently observed a similar mechanism in chickpea (*Cicer arietinum* L.) plants inoculated with *Fusarium oxysporum* f.sp. *ciceris* (*Foc*) under water stress, which resulted in higher photosynthesis and higher chlorophyll accumulation when compared to those of pathogenic inoculation or water stress treatment (Figure 1, unpublished data; the photograph of chickpea was used with the courtesy of Ummuhan Kasikci Simsek from her PhD study). Combinations of pathogens and water shortages caused a “sudden death syndrome” within a few days, after which this picture was taken. Therefore, we could easily state that the combined effects of drought and pathogen stress are more devastating to crop plants than either stress factor alone. Although antagonistic interactions are uncommon, either stress factor (drought or pathogen) could have priming effects on plants. However, this may not last long enough to trigger the defense mechanism. There are a few reports linking viruses to high-temperature or drought stress conditions [9,10].

In very rare cases, we could use mild pathogens to trigger defence mechanisms before drought stress occurs. With this approach, crop plants may synthesize drought- or temperature-related proteins or carbohydrates to tolerate stress [8,11]. For example, Xu [12] stated that increases in glucose, fructose, and sucrose levels in virus-infected plants might play significant roles as osmoprotectants. Induction of abscisic acid (ABA) upon infection, followed by viral attack, could lead to stomatal closure, thereby maintaining drought tolerance. Also, metabolic activities associated with viral infections might play protective roles, such as osmotic adjustment and membrane stabilisation. On the other hand, some metabolites are maintained at high levels under water stress; thus, plants may exhibit improved tolerance to virus infection upon virus attack. Prakash et al. recently stated that the host machinery could be redirected by the virus, and its cellular resources are allocated to combat drought stress [13]. They speculated that viral pathogens might shift from parasitism to mutualism, thereby activating host processes that promote drought tolerance. However, most of these findings have been reported with virus infections under drought conditions. Only one report from bacterial studies was made in that *Pseudomonas syringae* pv. tomato DC3000 propagation in *Arabidopsis thaliana* (L.) Heynh. was reduced, followed by drought stress [Gupta et al. 2016] [14]. Combined stressed plants displayed reduced reactive oxygen species (ROS) generation and declined cell death. The authors stated that endurance was provided toward the oncoming pathogen infection. However, there are more complex pathways than we previously thought for generalizing the antagonistic effects between abiotic and biotic stressors. It is not clear why some interactions result in tolerance while others lead to susceptibility. To elucidate and reveal the detailed mechanisms of plants under the combined stress, the plant stage, species, duration of each stress factor, and the characteristics of the stress, whether it is simultaneous or sequential, should be well established.

Although research has been conducted for many years, the mechanisms of interactions under multiple stress conditions remain poorly understood. Understanding how pathogens respond to drought stress in vitro, *in vivo* or *in planta* is crucial for enhancing stress tolerance in agriculturally essential crop plants [15]. In this review, we evaluated the combined effects of the foliar pathogen *Zymoseptoria tritici* and drought stress on cereal crops from multiple perspectives, including additive, synergistic, and antagonistic mechanisms, and discussed potential remediation and improvement strategies.

Drought stress, whether it adds to or stimulates pathogenic effects, could increase the pressure on us to produce high-quality wheat in semi-arid and arid areas.

To elucidate distinct pathways, we used “resistance” or “resistant” for pathogens, and “tolerance” or “tolerant” for drought stress issues.

## 2. Concept of Drought and Pathogen Stress

Under projected climate change, drought is one of the most critical abiotic stresses that severely affects agricultural crop development.

Abiotic stress factors have been more common than biotic stress factors in the last three decades. Drought and temperature stresses have become predominant in many parts of the world, even in moderately cool zones [16]. These stressors severely reduce crop quality and quantity. Global climate change and rising temperatures can affect the moderately cool climatic zones that were previously considered safe. Therefore, drought stress has even become more intense in these regions due to irregular rainfall, along with improper irrigation systems [17]. Quantifying the exact contribution of drought stress to global and even regional crop yield losses is complex because other stressors are involved. According to recent reports, abiotic stresses in total can account for up to 70% of yield reductions in staple food crops worldwide [18,19]. Drought, in particular, accounts for a significant portion of crop yield losses (70%) across all abiotic stress factors considered [20]. In low and middle-income countries, drought stress accounts for almost €40 billion losses [21]. Since drought stress accounts for the largest share of stress factors globally, we need to underscore its effects and interactions with other abiotic and biotic stressors. Drought stress is particularly severe during the vegetative and generative growth stages of crops that require substantial water, prolonging the germination period and delaying sowing. Unfortunately, the sowing date has been continuously postponed in the autumn season in the northern hemisphere due to severe drought. When water becomes available, followed by precipitation towards the winter season, the air temperature and daylight decrease drastically. Therefore, drought stress not only affects crop plants but also interacts with other stressors.

Drought stress basically causes loss of turgor pressure, decreases photosynthetic activity, and wilting of leaves due to plasmolysis, reduces vegetative growth, and causes cessation in both shoot and root development [22]. Additionally, it can decrease leaf area and the number of leaves. These changes can result in leaf shedding, reduced transpiration rates, decreased stomatal conductance, increased leaf temperature, disruption of the cell membrane system, reduced chlorophyll content, and complete plant desiccation during prolonged drought periods [23]. It can also affect the survival and spread of pathogenic microorganisms, although findings about the virulence levels are controversial [24]. We are aware that tolerance levels of microorganisms to abiotic stress factors are generally higher than those of many crop plants [2,9]. If a microorganism can survive in adverse conditions, it grows and sporulates efficiently and may maintain its pathogenicity, although its virulence may be altered [25]. Under these circumstances, plants would face more than two stressors: pathogens and drought, and their combined effects. Under combined stress conditions, we observed either synergistic or additive effects of stress factors in agricultural lands [26]. The stress that occurred following the combined stress could cause more severe symptoms of one of the stress factors, or it could be different from all the stress factors involved. In that case, the symptoms may be unique and should be characterized differently. The behaviours of plant pathogens, particularly *Z. tritici*, need to be evaluated in terms of their physiological, biochemical, and molecular pathways under drought stress conditions to generate multiple stress-tolerant cereals. An understanding of the combined stress mechanisms of drought/high temperature and of wheat pathogens would be a good model for other abiotic stress tolerance in cereal plant species. For example, if a pathogen can survive and express its pathogenicity under drought conditions, it should be controlled only by more resistant and tolerant plant varieties or by biocontrol agents that can survive and propagate under drought. For example, Bois et al. [27] stated that fungi were quicker to accumulate osmoprotectants, as well as Na^+^ and Cl^-^ ions, which might be deleterious for their hosts in the long term. Again, Pal et al. [28] reported that mild drought stress increased *Xanthomonas oryzae* pv. *oryzae* infection irrespective of the genotype of rice. Water deficits accelerated and exacerbated Pierce’s disease (PD) of grapevine (*Vitis vinifera* L.) symptom development caused by *Xylella fastidiosa* (*Xf*) [29]. The symptoms of PD are qualitatively and quantitatively distinct from those of water deficits. To evaluate the aetiology of the symptoms, grapevine plants were either exposed to water stress or inoculated with *Xf*. The infected watered plants exhibited symptoms unique to PD, such as green islands in stems, leaf blade separation from the petiole, and irregular leaf scorch. Ramegovda and Senthil-Kumar [30] reported that different signaling pathways occurred during the combination of abiotic and biotic stress occurrences. In this concept, Sartori et al. [31] showed that *Bacillus amyloliquefaciens*, a biocontrol agent, showed excellent tolerance at low water potential and high survival rates under heat stress against *Fusarium verticillioides* (Sacc.) Nirenberg. So, it would be more appropriate to search for microorganisms and evaluate their metabolic and genomic functions under drought stress to breed drought-tolerant plants and beneficial microorganisms. Nie et al. [32] reported that arbuscular mycorrhizal (AM) fungi had a significant role in enhancing plant growth and performance under environmental stress. Similar findings were made by Sunkad et al. [33] who stated that the dry root rot agent for *Macrophomina phaseolina* in chickpea plants was effectively controlled with *Trichoderma asperellum*. Apart from *Trichoderma* spp., *Clonostachys* spp. were also successfully used [34]. The authors stated that defence-related genes in wheat plants were strongly expressed at early time points (8 h) when wheat leaves were sprayed with *Clonostachys rosea* (Link) Schroers, Samuels, Seifert & W. The induced genes were associated with chitinases, oxalate oxidases, E3 ubiquitin-protein ligases, and protein kinases, which were validated in the defence response of wheat plants.

Some researchers used a multi-stress-tolerant rhizobacterial strain, *Bacillus subtilis* PM32, to control potato stem canker caused by *Rhizoctonia solani* Kühn [35].

In addition to biochemical, microbial, and molecular approaches, elicitors have also been suggested to enhance the defence mechanisms of wheat plants under stress. For example, the elicitor (surfactin) isolated from the strain *Bacillus amyloliquefaciens* s499 protected wheat by 70% against *Z. tritici* [36]. Although surfactin showed no antifungal activity against the pathogen, 23 defence-related genes in wheat plants were upregulated. The authors stated that surfactin significantly induced natural defences in wheat by stimulating both salicyclic acid (SA)-and jasmonic acid (JA)-dependent signaling pathways. It is clear that ABA suppresses SA and JA signalling pathways, thus the production of defence-related enzymes and metabolites. Therefore, activation of defence-related metabolites, especially those activated in both signaling pathways, is crucial for their activation under the combined stress of drought and *Z. tritici*. With these approaches, especially those that could be activated under the combined stresses, we could promote sustainable agricultural practices and reduce chemical inputs. High levels of ABA in plants can repress defence gene expression by suppressing the signaling pathways mediated by SA, JA, or ethylene (ET). For example, exogenous application of ABA on *Arabidopsis* plants increased the virulence of *Pseudomonas syringae* pv. Tomato [37]. Similarly, the application of ABA suppressed the transcription of defence-related genes in *Arabidopsis* plants against *Fusarium oxysporum* (causal agent of wilt) [38]. Again, tomato mutants deficient in ABA synthesis resulted in reduced growth of *Botrytis cinerea* [38]. These findings indicate that high ABA expression blocks the defensive pathway in plants. Although high ABA expression results in rapid stomatal closure, which hinders or prevents pathogen penetration, leaf pathogens can still penetrate the tissue. Fones et al. [39] reported that non-virulent *Z. tritici* strains proliferated epiphytically when they were unable to penetrate wheat leaves. The authors stated that these isolates later led to necrosis, and they might undergo sexual crosses with other isolates in the absence of leaf penetration.

By monitoring plant hormone pathways, pathogens can either suppress defence responses by increasing stress hormones to colonize plant tissues-, or hijack nutrients from hosts by increasing growth hormones to facilitate colonization and dissemination. It is clear that ABA suppresses the SA signaling pathway during severe drought, and therefore, PR proteins may be suppressed, resulting in susceptibility to biotrophic pathogens. Although stomata tend to close during drought, and pathogen entry may be expected to reduce, SA immunity weakens, and internal colonization increases. It has been commonly observed that if SA dominates, defence against biotrophs, if JA dominates, defence against necrotrophs occurs.

Although protective fungicides have been applied on many occasions, and the use of resistant varieties has been accepted as the most effective method, prolonged dry seasons would affect the behaviour of plant pathogens, crop yield, and quality. Dry seasons, followed by a lack of irrigation facilities, would force us to grow wheat under much harsher conditions. Yet the possibility of fungal adaptation to adverse conditions would threaten drought-tolerant and disease-resistant wheat plants. More stressful conditions for wheat growth would require scientists to develop plants that are more resistant and tolerant. However, achieving this would be costly, and we may have to sacrifice desirable traits to increase resistance or tolerance mechanisms.

We could evaluate the behaviours of *Z. tritici* under various drought scenarios. In a worst-case scenario, *Z. tritici* may not exhibit any decline in sporulation or mycelial growth, remain virulent and aggressive, and may continue to carry out its pathogenic activity. For example, our preliminary results (unpublished) showed that *Z. tritici* grown *in vitro* under various drought conditions that were simulated by using polyethylene glycol (PEG) exhibited a similar growth curve up to moderate drought levels when compared to that of the control group of *Z. tritici* grown under no drought conditions. In this scenario, drought stress may increase stress metabolite levels in wheat plants while decreasing antioxidant capacity and photosynthetic capacity. Stomata of wheat plants are closed to save water in cells at the cost of reduced photosynthesis. However, even if the closed stomata seem to prevent pathogen entry, *Z. tritici* could still penetrate the leaf cuticle and sporulate freely in internal tissues [40]. Many authors have stated that the cuticle was disrupted and thinned under drought stress conditions, thereby facilitating fungal penetration [14,25,41]. Defence signaling could also be impaired during drought stress. The closure of stomata, followed by drought stress, diverts the penetration of *Z. tritici* to the epidermis and cuticle. If the cuticle thickness is not strong enough to prevent or impede pathogen entry, then the pathogenic effects of *Z. tritici* become more pronounced, even if the fungus virulence is not increased. The increased pathogenicity under these circumstances could be attributed to reduced photosynthesis, which led to lower levels of pathogenesis-related (PR) proteins involved in preventing pathogen propagation [39]. In these circumstances, the pathogen appears more aggressive than it does. For example, Sharma et al. [42] and Fones et al. [43] reported that mild drought stress led to higher accumulation of fungal biomass than under well-watered conditions.

In a worst-case scenario, drought stress may stimulate mycelial growth and sporulation and increase pathogenic enzyme activity and melanin synthesis via virulence-related gene expression. Under slightly dry conditions, our preliminary results showed that sporulation of *Z. tritici* was even stimulated. Earlier, Guhr & Kircher (2020) [44] reported that drought stress exerted a priming effect on *Penicillium chrysogenum* sporulation and mycelial growth under *in vitro* conditions. The authors concluded that β-glucosidase activity played a significant role in the priming effect of drought stress [44]. We could increase the number of examples of fungal growth and sporulation under drought stress.

In a general concept, fungi tolerate abiotic stress much better than crop plants do. Drought stress tolerance in fungi should be considered carefully in light of these concepts. Therefore, the combination of *Z. tritici* and drought could have more devastating effects on plant health. It is highly crucial to understand the simultaneous or consecutive effects of the combined stress factors on plant defence mechanisms. Investigating the physiological and biochemical changes in plants under the combined stress of drought and pathogen attack can provide valuable insights into the synergistic or antagonistic effects of these stressors. We understand that the relationship between drought stress and the development of fungal pathogens is complex and can vary widely. Therefore, more targeted research is needed to draw definitive conclusions about the effects of drought stress on the sporulation and mycelial growth of specific foliar pathogens in wheat and other cereal crops, as well as in other crop plants.

The combined stress of drought and pathogen should also be evaluated under scenarios of sequential or simultaneous stress conditions. This is of great importance because sequential and simultaneous combined stresses have distinct mechanisms, as revealed in other plant-pathogen combinations [8,45]. Cereal plants can mostly face sequential stressors in nature. Although it is very rare, simultaneous stress factors can also significantly affect cereal responses. Whichever stress factor comes first must be dealt with first.

## 3. Effects of Drought on Cereal Cultivation

The importance of wheat is unquestionable, as it plays a vital role in global nutrition and human health. However, the cultivated areas of cereals, particularly wheat, are considerably smaller than in previous decades. According to FAO data, the harvested area of wheat in Türkiye reached approximately 9.74 million hectares in 1994, but declined to approximately 6.83 million hectares in 2023 and 6.93 million hectares in 2024. This represents an overall decrease of approximately 29% in wheat cultivation area over the past three decades [46]. Wheat is a widely cultivated annual plant across diverse climatic and soil conditions, with global production exceeding 840 million tons [47]. Drought stress could cause a 50% loss worldwide [48]. Biotic agents cause significant yield losses, accounting for 30–35% of wheat yield, particularly in winter varieties [49]. The loss caused by *Z. tritici*, in particular, not only has a significant economic impact but also threatens product quality. As global temperatures increase, the loss is becoming more significant [50]. Since this section has been evaluated many times in various studies, whether as reviews, research articles, or book chapters, we briefly summarized the negative impacts of drought on cereal cultivation.

## 4. Pathological Responses of Wheat Plants Infected with *Z. tritici*

Cereal plants face several pathogens during the seed, seedling, vegetative, and generative growth periods. The major diseases are leaf-borne and easily disseminated via air and agricultural equipment. The significant pathogens include *Tilletia* spp., *Ustilago* spp., *Puccinia* spp., *Zymoseptoria tritici*, *Drechslera sorokiniana*, *Fusarium* spp., *Rhizoctonia* spp., and *Sclerotium* spp. Among them, *Z. tritici*, one of the most devastating wheat pathogens, has been characterized by humid and cold conditions; however, the pathogen has now been considered as a stress-adapted fungus [1,51,52]. One of the key pieces of evidence supporting this view is the climate reports showing gradual temperature increases during the vegetative and generative wheat growth (April–August) period from 1981 to 2015 in the northern hemisphere [53]. The incidence of the fungus, in terms of pathogenicity and spread, has been increasing. Although high wheat-yielding cultivars have been used in many places globally, no significant declining trend in terms of *Z. tritici* infection has been evident [54]. High-yielding newly generated wheat species have been bred to optimize growth conditions and achieve higher crop yields per unit area. These cultivars mostly possess thin wax layers to use light more efficiently to increase photosynthetic capacity [55]. Even if a temporary or prolonged drought occurs in *Z. tritici*-infected wheat areas, interactions between the pathogen and drought, or with other abiotic stressors, are likely to happen. Therefore, potential interactions between the possible growth and dissemination of *Z. tritici* under drought conditions on wheat leaves with thin cuticle layers would facilitate pathogenicity by enabling easier penetration, while negatively affecting cereal growth and development.

*Z. tritici*, from the *Mycophaerellaceae* family of *Dothidiomycetes* class, causes leaf blotch and necrotic spots in wheat leaves. In these spots, the fungus forms perithecia (sexual) structures to initiate the infection cycle via production of ascospores, followed by pycnidiospores (asexual) in pycnidia to disseminate and spread the pathogen cycle [56]. The initial infection begins in late summer or autumn and spreads through the air. The secondary infections are then carried out in the spring season when spores are carried on leaves due to rain, dew, sprinkler irrigation, or fertigation [57]. Mycelia arising from spores are involved in penetration through the leaves, stems, and spikes of wheat plants Although the pathogen prefers to infect through the stomata, this is not always the case. Able to move by forming conidia or mycelium, *Z. tritici* can easily reach the stomata or penetrate any surface it encounters.

The pathogen overwinters in diseased plant residues, seeds, or infected plants as pycnidia or mycelium [58]. The pathogen is hemibiotrophic, meaning it acts as both biotrophic (obligate) and necrotrophic (facultative). The biotrophic stage begins with the formation of sexual spores [59]. The transition from the biotrophic to the necrotrophic stage is generally characterized by pycnidia formation [60]. In the necrotrophic stage, host defence is generally suppressed, and therefore, polycyclic behaviours of the pathogens begin. During this stage, nutrient search by the fungal mycelia increases, and more nutrients are released into the cell cavity [61]. Abiotic stresses such as salinity, drought, and temperature result in increased accumulation of low-molecular-weight carbohydrates and metabolites, such as glucose and its derivatives, and low-molecular-weight amino acids, to regulate osmotic potential. So, the accumulation of these compounds would encourage the spread and dissemination of the fungal mycelia [62]. For example, Dikilitas et al. [63] stated that a significant positive correlation was established between glucose, amino acid compounds, and enzymes such as protease, which were correlated with the virulence of *Pyrenophora teres* f. *maculata* (*Ptm*) and *Pyrenophora teres* f. *teres* (*Ptt*), the causal agents of spot and blight diseases in barley. *Z. tritici* encodes quite a few carbohydrate- and protein-degrading enzymes, such as laccase and protease [64].

The primary substrates, such as glucose and its derivatives, provide both energy and structural materials for plant defence responses and also function as signaling molecules that initiate these defence mechanisms. High sugar content enhances the oxidative burst in the early stages of infection, increases lignification of cell walls, and stimulates the expression of flavonoids and PR proteins [65]. However, high sugar levels do not always boost the defence system in plants; in fact, they stimulate sporulation and mycelial growth in fungi. Granda and Camarero [66] reported that drought reduced growth while stimulating sugar accumulation in trees. Sugar accumulation should not be considered just as a simple biochemical pathway in response to stress. There is a positive correlation between sugar accumulation and toxin production. For example, Arias et al. [67] reported that toxin-producing *Aspergillus flavus* synthesized laccase 300-fold in the presence of sucrose (3%), and 250-fold in low water potential (−1.1 MPa) when compared to control conditions upon infection of peanut invasion. The authors stated that high sugar contents or drought conditions could contribute to peanut infection at early stages of growth. Therefore, opportunistic pathogens, protease- and laccase-producing fungi, and toxin-producing fungi could pose a potential danger under drought conditions.

Small secreted proteins (SSPs) produced by the pathogen also facilitate infection. When the pathogen penetrates the cell, it moves from the apoplast (extracellular space) to the symplast (cytoplasm). During this stage, signaling molecules produced by the cell trigger defence mechanisms via synthesizing PR proteins, reactive oxygen species (ROS), and defence-related genes in a network concept [68]. At this stage, resistant wheat plants may synthesize more metabolites to combat the pathogen; however, any additional stress factors tolerated by the fungus would result in more devastating effects on wheat plants. The more metabolites accumulated by cereals on leaves would be used as substrates by the pathogen, as demonstrated by Rojas et al. [69]. Similarly, Dikilitas et al. [41] stated that tomato plants accumulated proline and protein metabolites to tolerate salinity stress, thereby providing a rich source for pathogen progression. For example, Dikilitas [70] reported that the pathogenic effect of *Verticillium albo-atrum* was more pronounced on lucerne (*Medicago sativa* L.) plants under salinity stress, and the pathogen was considered more virulent, although this was not the case.

The semi-obligate pathogen also possesses dimorphic life patterns. This means that the fungus switches between the yeast and mycelial forms under stress conditions. For example, increases in temperature from 18 to 25–28 °C transformed mycelial structure into yeast form [71]. However, temperature fluctuations may not be sufficient for dimorphism, since transitions were not observed or reported under heat stress in many parts of the world [72]. The transition between yeast and mycelial growth is reversible; therefore, factors such as nutrient availability, pH and temperature stress, host behaviour, and growth conditions can significantly affect this transition. For example, during early infection, a yeast-like state can be observed, but during colonization and a necrotrophic state, filamentous growth is observed to search for nutrients in leaf cells. Therefore, solid surfaces such as leaves, solid media, and nutrient deprivation mostly encourage filamentous growth [73]. Francisco et al. [74] reported that signaling systems of the fungus were more efficient during mycelial growth in stress conditions than those of yeast-like growth. With this pattern, the stress response was much quicker. Therefore, we need to identify the transition pattern between yeast-like and mycelial growth under other stress factors, such as temperature and nutrient deprivation, to breed more resistant wheat cultivars. Releasing the transition pattern of *Z. tritici* on wheat plants under drought-stress conditions could either encourage or discourage the transition, benefiting cereal growth. For example, by extending the yeast-like growth period with chemicals or abiotic elicitors, we could reduce the impact of the pathogen. The vegetative growth stages of *Z. tritici* are divided into three groups. Macro- and micro-pycnidiospores and unicellular forms. Macro- and micro pycnidiospores produce hyphae and are found in dormant pycnids; the unicellular group turns into a yeast-like form [75]. Macro and microconidia are crucial agents in the initiation and dissemination of the fungus. *Z. tritici* is a strictly apoplastic fungus. Rebaque et al. [76] stated that *Z. tritici* secreted cell wall-degrading enzymes (CWDES) facilitated colonization; however, damaged or infected cells through cell wall-derived oligosaccharides triggered host immunity. They suggested that cell wall degradation by fungal CWDES and the release of immunogenic wall-derived oligosaccharides regulate the outcome of host invasion by pathogens. The authors revealed that β-1,3/1,4-glucan oligosaccharides produced during *Z. tritici*-wheat interactions served as danger signals that triggered wheat immunity, leading to the production of ROS, stomatal closure, and a transcriptional response. During the necrotrophic phase, disease severity and sporulation levels were associated with β-1,3-glucanase and protease [77]. Meile et al. [78] reported that *Z. tritici* emerged during the domestication of wheat in the Fertile Crescent in the Middle East. Tian et al. [79] reported that fungal chitin triggers the activation of immune responses in wheat plants upon recognition. To avoid immune responses, *Z. tritici* secretes LysM effector proteins. The authors stated that Mg1LysM and Mg3LysM effector proteins protected fungal hyphae against host chitinases. The authors reported that Mg3LysM conferred a major contribution to *Z. tritici* virulence. They suggested that *Z. tritici* utilizes LysM effector proteins to disarm chitin-triggered wheat immunity. This disease affects all regions across Central Europe, Asia, the Mediterranean region, and the Americas. Epidemics occur in wet years. Although *Z tritici* is a hemibiotrophic pathogen, it is mostly characterized by a long biotrophic stage in plant tissues [80]. Francisco et al. [74] discovered that *Z. tritici* forms chlamydospores that survive in extreme cold and drought conditions. They stated that the fungus’s possession of different mycelial and spore types, such as blastospores, pycnidiospores, and chlamydospores, may help it survive stressful conditions and regulate morphogenesis and virulence. The authors speculated that the extent of morphological changes increases the adaptive capacity of the fungus to different environmental conditions. It has been reported that blastospores of *Z. tritici* can survive in water or soil for extended periods [81]. The authors also stated that the fungus most probably uses lipid stores in their cells. They claimed that the virulent isolates most probably grow on rich media. Water film on the leaf surface not only supports pathogen multiplication but can also sustain microbial pathogens.

The first symptom of *Z. tritici* in wheat plants starts with the formation of chlorotic spots on the lower leaves. The spots are irregular with a pale yellow colour at the beginning, which could be clearly distinguished from a distance. As the disease progresses, these spots turn black with grey heads in the leaf. These spots contain pycnidia, which disperse under humid conditions.

Fungicides have largely controlled the disease, but pesticide resistance has hindered pathogen spread. Despite frequent applications in many locations, virulent pathogenic races have outpaced the toxic effects of pesticides [59]. Yet, in most cases, fungicide applications did not control the disease [82]. Environmental concerns are another issue to consider, putting additional pressure on growers and consumers seeking high-quality crops. For example, Garnault et al. [83] reported that *Z. tritici* in French wheat fields showed resistance to recently developed pesticides, including benzimidazoles, quinone outside inhibitors, sterol demethylation inhibitors, and succinate dehydrogenase inhibitors. Hayes et al. [84] stated that fungicide resistance was evident in *Z. tritici* due to frequent fungicide applications to control new, aggressive populations of wheat stripe rust.

Although resistant varieties may help, annual crop loss remains high worldwide [85,86]. However, even using resistant varieties may not help if abiotic stress is involved. For example, Bartosiak et al. [87] reported that *Z. tritici* could adapt to high temperatures and other abiotic stressors. We could be optimistic if cereal cultivars exhibiting abiotic stress tolerance show resistance to *Z. tritici* infection. Recently, Baran et al. [1] stated that a salt-tolerant wheat cultivar (Ceyhan-99) showed better resistance to *Z. tritici* infection than a salt-susceptible (Nurkent) cultivar. A positive correlation between abiotic stress tolerance and *Z. tritici* resistance could be a promising approach for generating multiple stress-tolerant cereal plants. However, this study needs further elucidation to clarify the mechanism. A similar connection was found between drought-tolerant chickpea (WR-315) and *Fusarium oxysporum* f.sp. *ciceris* by Simsek et al. who stated that the drought-tolerant chickpea cultivar showed better resistance to *F. oxysporum* infection than that of the drought-susceptible cultivar. It is evident that fungi can produce metabolites under adverse conditions [82,88]. Therefore, a combined stress is inevitable even if synergistic effects are not observed.

**Figure 2:**
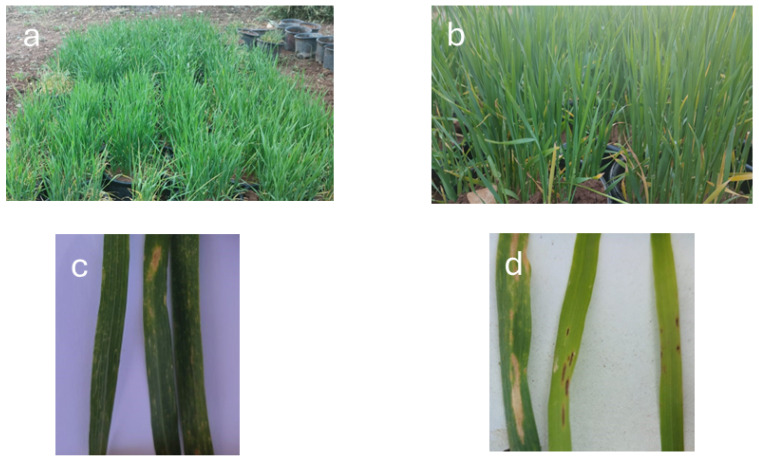
Responses of wheat plants towards drought, *Zymoseptoria tritici* infection, or both in field conditions. (**a**) Control plants; (**b**) symptoms of drought stress; (**c**) pathological symptoms of *Z. tritici*; (**d**) the combined effects of drought and pathological symptoms on wheat plants.

The detailed infection mechanisms of *Z. tritici* were explained in Baran et al. [1]. *Z. tritici* causes characteristic symptoms on wheat leaves; however, under drought stress, the symptoms can be more severe (Figure 2).

Drought stress reduced chlorophyll content and led to chlorosis, while only *Z. tritici* infection resulted in characteristic spots and blotches along leaf stripes. The combined stress of drought and pathogen exerted much more severe symptoms than either stress factor alone. As the duration of stress increases, it can become quite difficult to distinguish the symptoms individually, as they become intertwined. Under these circumstances, we could interpret the dominant stress symptom as a much more severe condition of a suspected stress factor.

## 5. Effects of Drought on the Metabolism of *Z. tritici*

Many plant pathogens have now been forced to grow, survive, and complete their life cycles under the adverse conditions due to environmental pollution and global warming. *Z. tritici*, with no exception, has been facing salinity and drought stress in nature. For example, Liu et al. [89] reported that a host-specific toxin, SnTOX1, obtained from *Parastagonospora nodorum* *(Berkeley) Quaedvlieg*, *Verkley & Crous* retained activity only above 50 °C under *in vitro* conditions. The biochemical or molecular behaviour of *S. nodorum* was like that of *Z. tritici*. Therefore, one should expect similar responses from *Z. tritici* regarding toxin and enzyme production under stress conditions. This clearly showed that *Z. tritici* could exhibit responses similar to those of other metabolite-producing fungi under stress conditions and that the fungus becomes very difficult to control, possibly adapting to stress conditions by producing osmolytes. We have recently proved that *Z. tritici* has a very strong potential to adapt to saline conditions [1]. Again, Mirzadi Gohari et al. [90] showed that a group of genes, *TOX2*, responsible for the toxicity in *Cochliobolus carbonum*, were also expressed in *Z. tritici.* Therefore, common biochemical and molecular mechanisms, at the metabolite and gene levels, across fungi could lead to similar responses and adaptations. Protein synthesis under normal conditions could occur during the penetration stages under drought stress, provided the fungus can grow under drought stress. For example, the protein Mg3LysM, which plays a significant role in disrupting the host immune system under drought stress, was produced or synthesized by the *Z. tritici* fungus [79]. So, metabolite functions and gene expression during host–pathogen interactions should be tested and elucidated in detail during stress conditions to determine the potential behaviour of the fungus. Since *Z. tritici* possesses sophisticated physical and biochemical weapons during penetration, pathogenicity, incubation, and sporulation, it can efficiently utilize complex organic compounds. Controlling this pathogen is, therefore, highly difficult. Low-molecular compounds rapidly degraded from complex carbohydrates and proteins under drought or high-temperature stress conditions to maintain osmotic balance would be used as substrates, thereby increasing the pathogen’s dissemination and spread and contributing to the fungus’s pathogenicity. Once a fungus starts producing pathogenic enzymes, it is very difficult to reduce the level of pathogenic enzymes thereafter [51].

Abiotic stress conditions may also influence fungal infection processes. For example, Somai-Jemmali et al. [77] showed that *Z. tritici* readily penetrated the susceptible host because the defence mechanism mediated by PR-protein-encoding genes was not established at earlier stages under drought stress. Drought stress may disrupt the mechanisms of cell defence systems and photosynthetic activity; therefore, we could easily speculate that *Z. tritici* would prefer to penetrate the thin cuticle layer rather than moving towards the stomata [91]. Under stress, *Z. tritici* produces stress metabolites compared with normal growth conditions. One of the stress metabolites highly expressed during stress is melanin. Since this metabolite was directly correlated with virulence and pathogenicity and was involved in neutralising ROS produced by the host, it may also play significant roles during drought stress by facilitating conidial adhesion to the host cell wall [92]. Kilinç et al. [52] reported that melanin was accumulated progressively up to 30 °C in *Z. tritici*. The authors stated that increases in melanin concentration were considered as a response to temperature stress. During this period, the accumulation of low-molecular-weight organic compounds, such as carbohydrates, proteins, and plant-derived phenolic compounds, was efficiently utilised by *Z. tritici.* They suggested that these metabolites were considered good substrates for invading pathogens. Tiley et al. [93] characterized a photoreceptor gene, *ZtWco-1*, in *Z. tritici*, which might be involved in the detection of light. The authors stated that this gene was a putative homolog of the *wc-1* gene of *Neurospora crassa*, acting as the blue-light photoreceptor. The authors suggested that deletion of *ZtWco-1* gene resulted in decreased hyphal branching, melanization, and virulence on wheat. Choi et al. [94] also identified a gene, *MVE1*, in *Mycosphaerella graminicola* associated with multiple signaling pathways, light detection, and melanin production. Induction of osmotic stress or low temperature resulted in downregulation of this gene. So, we could say that light detection and melanin synthesis are linked and play significant roles in fungal virulence. Light wavelength and quality could also affect the fungal development of *Z. tritici*. It was reported that the blue light significantly reduced colony growth while the red light favored fungal development [95]. McCorison and Goodwin [96] revealed that *Z. tritici* sensed and responded to light, which could be important for pathogenicity. However, it is not clear which color of light stimulates the sporulation of *Z. tritici*. Toledo et al. [97] reported that melanins were associated with stress tolerance, virulence, and energy transduction. Aggwarwal et al. [98] reported a significant positive correlation between sporulation and melanin content in *Bipolaris sorokiniana* (Sacc.) Shoemaker infecting wheat. Similar findings were also made by Bashyal et al. [99]. Melanin accumulations also played significant roles in human pathogens. For example, Nosanchuk et al. [100] reported that melanin significantly increased the virulence of many important human-pathogenic fungi.

Drought stress not only leads to the accumulation of low-molecular-weight sugars and amino acids that promote the growth and sporulation of the invading fungus but also induces stomatal closure via ABA synthesis to prevent water loss. However, this would reduce the capacity of photosynthesis and energy production to generate sufficient ATP and PR proteins that would otherwise confound the pathogen; therefore, progression of the pathogen within the tissue due to limited energy production by the host plant would eventually result in host susceptibility [101].

**Figure 3:**
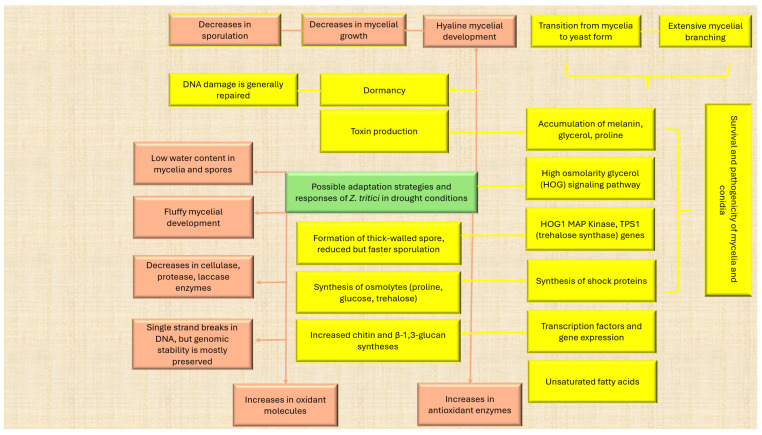
Possible responses and adaptation strategies for wheat leaf fungi (*Z. tritici*) under drought conditions.

Fungi use various adaptation strategies through biochemical and molecular mechanisms. Adaptation strategies of leaf fungi to drought conditions show more or less similarities to those of root pathogens (Figure 3). We summarized the various metabolites and mechanisms that *Z. tritici* could potentially use to tolerate drought stress.

Turco et al. [102] reported that mycelial growth of *Seiridium cardinale* (W.W. Wagener) B. Sutton & I.A.S. Gibson, *S. unicorne* and *S. cupressi* decreased with decreasing water potential and ceased at −15 MPa, although the mycelium remained alive. Histochemical analysis revealed that −12 MPa resulted in melanization and thickening of hyphal walls. Water potential around −3 MPa stimulated *S. unicorne*, indicating that drier conditions in Mediterranean ecosystems due to global warming would increase the adaptive potential of the fungi. This clearly shows that fungi, in general, perform better under drought stress than wheat plants. Palmero Llamas et al. [103] also demonstrated that marine isolates of Fusarium solani developed a physiological mechanism enabling survival in environments with low water potential. We could state that prolonged drought stress or short-term water stress may have different effects on fungi than on crop plants, with fungi showing signs of stress rather than adaptation. As stated before, fungi could survive in extreme conditions where most crop plants cannot grow even if they can germinate. So, biocontrol of fungal agents that confer pathogenicity should be achieved with microorganisms that can withstand extreme conditions. For example, *Verticillium lecanii*, used as a biocontrol agent, could survive at a water potential of −12.3 MPa. At this concentration, no agricultural crops were reported to survive [104]. The authors stated that fast germination and growth under severe drought conditions could be a desirable criterion for biocontrol agents. We know that low levels of drought stress stimulate fungal germination, sporulation and mycelial growth, where most crop plants show signs of osmotic stress. Therefore, fungi that could be used as biocontrol agents should also carry these characteristics. For example, Chandler et al. [104] reported that germination of *Verticillium lecanii* was rapid at −0.2 MPa.

*Z. tritici* can produce metabolites to sporulate and promote mycelial growth. Fungi experience water limitation, osmotic stress, and metabolic imbalance under drought stress. Under drought, fungal cells lose water and turgor pressure. To maintain intracellular osmotic balance, fungi synthesize compatible solutes such as glycerol, trehalose, mannitol, and proline. These osmolytes help cells maintain turgor by stabilising proteins and membranes, thereby preventing or mitigating dehydration-induced damage. Glycerol accumulation helps the pathogen survive on dry wheat leaves. By synthesising chitin and glucan, *Z. tritici* tend to lose less water and increase mechanical stability. Synthesis of unsaturated lipids during drought stress maintains membrane stability, while antioxidant enzymes play a great role in removing ROS. Metabolic shifts between TCA and glycolysis may play significant roles in energy supply. Under drought stress, energy metabolism shifts toward survival. If these metabolites are produced in sufficient quantities or concentrations, this strategy could easily lead to drought adaptation of the fungus. When *Z. tritici* adapts to drought through the mechanisms above, it may still exhibit pathogenicity. Decreased mycelial growth, faster sporulation, and increased enzyme synthesis are still sufficient to impair host defence by increasing cuticle penetration and toxin production. This could be reflected as enhanced infection under drought stress. Metabolites in yellow boxes connected by yellow lines indicate adaptation strategies, while red boxes connected by red lines indicate the instant responses of the fungus. Some common mechanisms of drought stress were adopted from other leaf fungi when *Z. tritici* was not available. The illustration is extensively modified from the works of Hohmann [105], Fillinger et al. [106], and Baran et al. [1], Suzuki et al. [107].

## 6. Combined Effects of Drought and *Z. tritici* on Wheat Plants

Many studies have focused on salinity and disease interactions in crop plants; however, pathogens can also interact with other abiotic stressors. Most importantly, leaf pathogens have recently been considered as they could interact with the abiotic stress factors. Previously, Baran et al. [1] showed that *Z. tritici* interacted with varying salinity levels in wheat plants, resulting in more severe symptoms and faster disease progression than pathogen inoculation alone. It is now firmly established that the combination of abiotic and biotic stressors exerts greater stress than either stressor alone [108]. Under a single stress factor, defence or tolerance mechanisms can be quickly activated, as can gene expression and biochemical responses. Stress-related compounds, phytoalexins, reinforcement of plant cell walls, gene expression, stress-related enzymes, and hormones can be efficiently induced, depending on the duration of stress and plant capacity [109]. However, when plants face dual stress, especially abiotic and biotic stress, they must follow two distinct pathways: the glycolytic and pentose phosphate pathways [110]. For example, Cao et al. [111] indicated that biotic and abiotic stresses might activate distinct Ca^2+^-permeable channels. They speculated that the calcium signal induced by biotic and abiotic stresses was independent.

**Figure 4:**
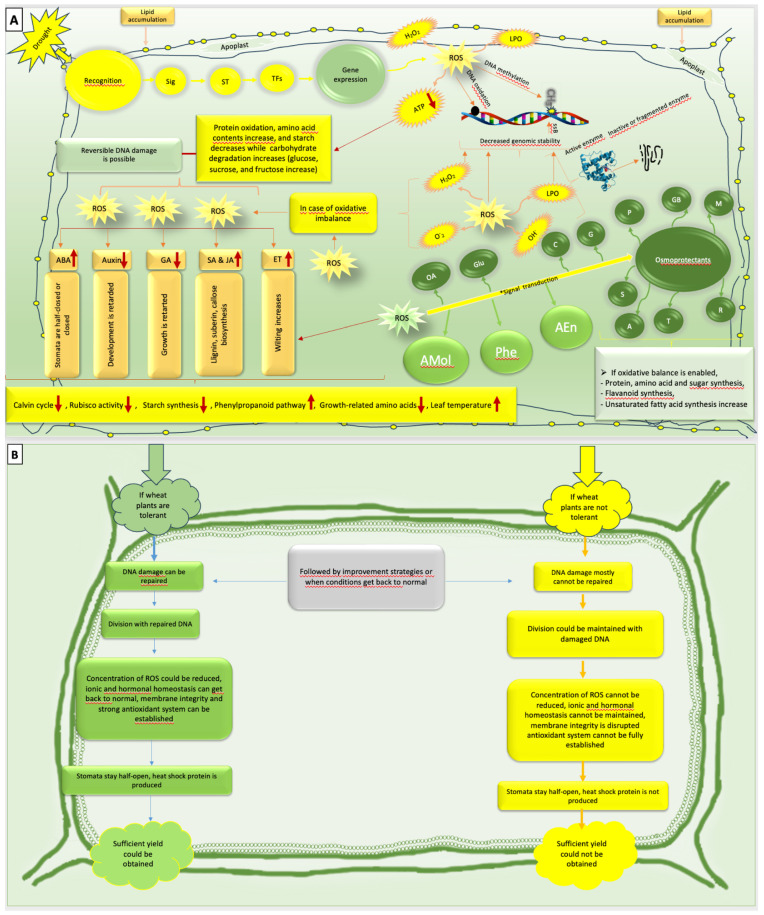
Cell metabolism of wheat plants under drought stress. (**A**) This section illustrates the cellular and molecular mechanisms of drought stress in plant cells, delineating the signal transduction pathways, hormonal fluctuations, reactive oxygen species (ROS)-induced oxidative damage, and the subsequent activation of osmoprotectant systems. (**B**) This section comprehensively compares the differential physiological and cellular responses of drought-tolerant and non-tolerant wheat varieties under water deficit, detailing their respective DNA repair capacities and the ultimate impact on crop yield.

**Figure 5:**
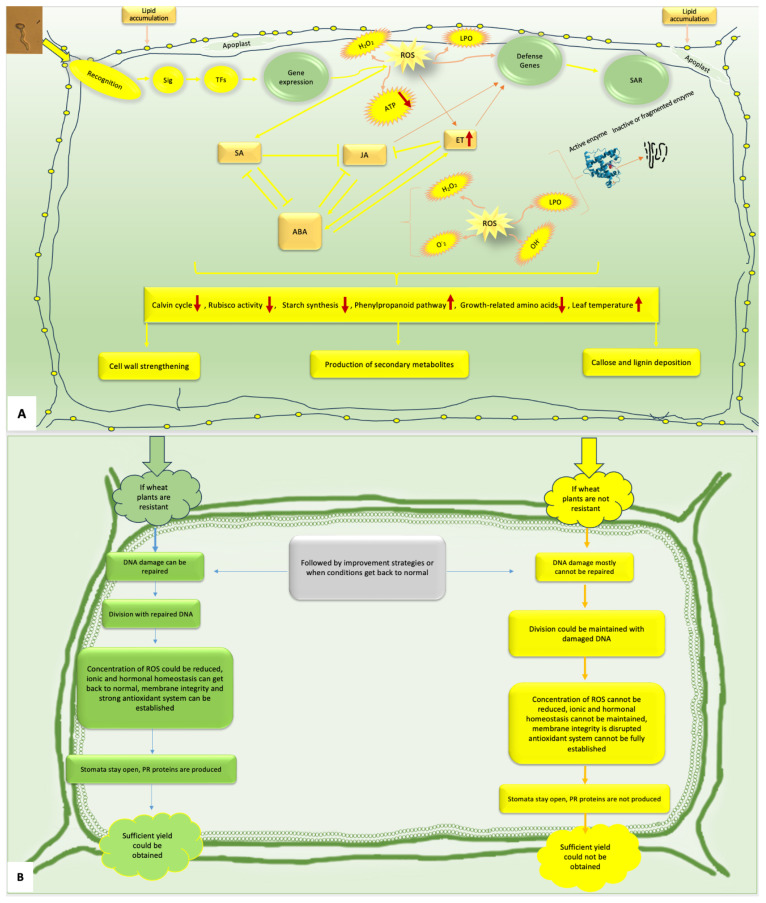
Cell metabolism in wheat plants following *Z. tritici* infection. (**A**) This section illustrates the intracellular signaling cascades, hormonal crosstalk, and reactive oxygen species (ROS)-mediated macromolecular damage triggered by environmental stress, alongside downstream metabolic adaptations including cell wall strengthening and secondary metabolite production. (**B**) This section delineates a comparative overview of the differential cellular responses between stress-tolerant and non-tolerant wheat genotypes under adverse conditions, contrasting their DNA repair efficiency and homeostatic maintenance capacity which ultimately dictate crop yield.

**Figure 6:**
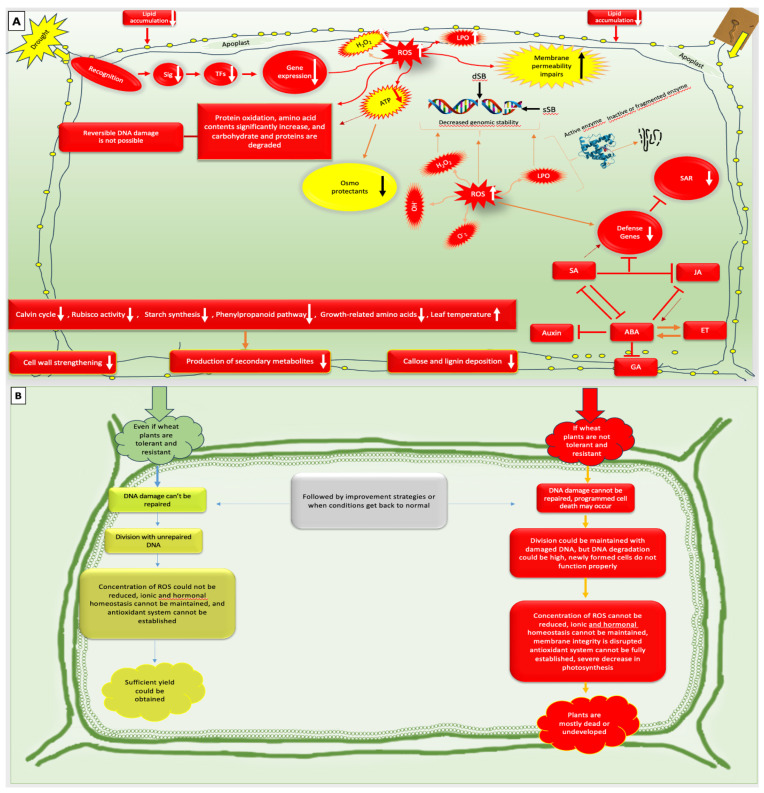
Cell metabolism of wheat plants inoculated with *Z. tritici* under drought stress (**A**) The upper panel illustrates the severe intracellular disruptions triggered by stress, characterizing the suppression of signaling cascades, elevated reactive oxygen species ($ROS$) accumulation leading to impaired membrane permeability and genomic instability, down-regulation of defense genes, and the consequential inhibition of metabolic adaptations such as cell wall strengthening and secondary metabolite production. (**B**) The lower panel presents a comparative framework of the downstream pathological consequences in wheat varieties under unmitigated stress, delineating how irreversible DNA damage, disrupted hormonal homeostasis, and failed antioxidant defense systems severely restrict physiological functions, ultimately compromising survival and crop yield.

These two distinct pathways should be alerted to stressors. However, this is very costly and signaling mechanisms should trigger defence tolerance pathways for both stress factors. For example, plants should synthesize compounds to mitigate the pathological stress factor while regulating osmotic and nutrient imbalances to mitigate the abiotic stress factor. Especially in drought or high-temperature conditions, water regulation and water uptake are severely disrupted. Even resistant or tolerant plants might have difficulty coping with combined stress, as it significantly alters plant defence metabolism. The detailed mechanisms of plant cell responses and pathways under single- or combined-stress conditions were illustrated in Figure 4, Figure 5 and Figure 6. Here, we focused, in particular, on the combined effects of drought and *Z. tritici* infection on wheat plants. The characteristic symptoms of drought, *Z. tritici*, and their combined stress on cereal plants are summarised in Table 1. The virulence or pathogenicity of the fungus may increase if it can tolerate drought stress. In most cases, sporulation or mycelial growth may decline; however, the amount of mycelia and conidia produced during drought stress may still be sufficient to initiate infection [112]. For example, Fones et al. [39] showed that successful infection can occur at very low spore densities. Recent works have supported our view. Based on our experience and scientific evidence, we do not expect any cereal plants to tolerate drought stress to the same extent as airborne fungi. Several publications support this view [112,113,114,115]. Very low drought intensity or scattered drought during the wheat growth period may increase sporulation and mycelial growth; even if it does not decrease sporulation, it may still contribute to pathogen dissemination. This scenario could be interpreted as synergistic, given the stimulation of fungal growth. If the combined stress factor follows a shared pathway for abiotic and biotic stressors, we may expect crop plants to spend less energy than plants following different stress pathways. Drought and pathogens are two different, distinct stressors. Plants have to close down the stomata; at least partial closure of stomata would be expected due to the accumulation of ABA upon exposure to drought, while defence-related metabolites, enzymes, proteins, and low-molecular-weight antimicrobial molecules, such as vitamins and phytoalexins, need to be produced to prevent or stop the sporulation and pathogenicity of the invading fungus [116]. It is clear that high accumulation of ABA, which is a stress hormone, impacts the pathogenicity and, therefore, devastating effects of the combined stress would be inevitable [117,118]. To cope with the effects of pathogens, drought-stressed plants should produce PR proteins to minimize pathogen damage [119]. So, the energy required to cope with the pathogen should be sustained with the products of photosynthesis. As explained above, under drought conditions, the photosynthetic efficacy of plants is greatly reduced. Therefore, the negative effects of the pathogen or drought would be expressed at much higher levels. So, the plants under drought stress are unlikely to share the same metabolic and defence pathways. Signaling cross-talk due to the incompatibility of different pathways may increase stress metabolites in plants and greatly increase energy consumption. For example, Satapathy et al. [120] reported that aluminium toxicity and *Fusarium incarnatum*-*equiseti* infections significantly increased ROS accumulation, cell death, and stress-related enzyme activity in *Cajanus cajan* (L.). Millsp.

Under moderate drought stress, pathogen propagation could lead to infection. In severe drought, the pathogen could still grow and sporulate, although sporulation is greatly reduced. Respiration increases, solute leaks, antioxidant molecules are depleted, and photosynthetic activity is impaired. Cytoplasmic viscosity decreases. As lipid molecules accumulate, membrane rigidity increases. Osmoprotectants regulate cell turgor, stabilize proteins, and buffer ROS. ROS could act as a signaling molecule when synthesised at early stages of stress occurrence, and it may result in the synthesis of antioxidant molecules and metabolites. Changes in the equilibrium between antioxidant and oxidant balance decide which way plants go. Under stress, oxidant metabolites tend to increase, while detoxification mechanisms reduce them. The phenylpropanoid pathway is activated, and defence-related metabolites tend to increase, such as phenylalanine, cinnamic acid, flavonoids, and lignin precursors. Mitochondria, chloroplasts, and peroxisomes are the main sites of ROS production, other than the cell membrane. Their accumulation leads to the oxidation of proteins, enzymes, and DNA, thereby modifying pathways and even disrupting cell membrane structure. Since major antioxidant- and defence-related enzymes take place in those organelles where many functional metabolic activities (ATP synthesis, chlorophyll production, respiration, degradation of organic acids, toxic metabolites) are carried out, stress metabolites such as O_2_^−^, O^−^, OH^−^, H_2_O_2_, and MDA directly affect the performance of those enzymes. Any additional stress molecules arising from additional stress factors could have devastating effects on organelles and DNA. When enzymatic and hormonal homeostasis are perturbed, ROS become highly toxic to cells and cell organelles. DNA damage is inevitable when antioxidants are insufficient to maintain equilibrium between ROS and the antioxidant system. Signal transduction leads to the accumulation of transcription factors (TFs), gene expression, enzyme activation, accumulation of antioxidant metabolites, and osmolytes to improve cellular conditions. If sufficient accumulation and activity occur, the repair process, through the reconstruction or restructuring of the plasma membrane, could reduce electrolyte leakage across membranes and regulate DNA repair and other cellular metabolic processes. This could enable drought tolerance or adaptation. Cell division could be regulated by reversible DNA damage, and ROS levels could be reduced by exogenous antioxidant application. A sufficient crop could be obtained. *To avoid confusion, we classified plants exposed to drought stress as “tolerant” or “non-tolerant,” and plants inoculated with *Z. tritici* as ‘resistant’ or “non-resistant”.

*Z. tritici* generally uses stomata for entry; possible reduced early infection could be observed under severe drought. However, necrosis may accelerate upon depletion of antioxidant molecules and increased ABA expression, which suppresses defence signalling. Due to necrotic lesions on leaves, photosynthetic capacity is reduced. Carbon metabolism is redirected; the plant focuses on producing defence-related compounds. Under pathogen stress, nitrogen uptake is disrupted, and metabolite quality shifts toward defence-related metabolites.

In general, stress sensing follows a known pattern summarized in the figure. There is no difference between abiotic and biotic stress in terms of stress responses. The main issue here is to decide which one started first and, therefore, which stress factor is sensed earlier. This actually determines the stress pathway. If the pathogen can survive and disseminate under drought stress, it can freely synthesise enzymes and toxins due to impaired host defence mechanisms. Organic materials degraded into sugars and amino acids, acting as osmolytes, would be exploited by the pathogen upon penetration through the stomata or cuticle. Following drought stress, the fatty acid composition of the cell membrane is altered, rendering it a favourable substrate for pathogen development. Drought stress disrupts the cell wall structure and predisposes wheat plants to pathogenic attack. Although ABA is involved in stomatal closure to prevent water loss under drought stress, overproduction of ABA could suppress defence responses by inhibiting the JA/SA pathway, thereby reducing systemic acquired resistance (SAR). Therefore, increased sporulation and pathogen dissemination have been commonly observed. Suppression of hormones such as ET, in addition to JA/SA, modifies photosynthetic and respiratory pathways, particularly the ATP pathway. Under drought stress and pathogen infection, ion homeostasis and most defence-related genes are downregulated, and defence-related metabolites are either not expressed or contribute only minimally to defence mechanisms. The outcome of this stress could be intensified by the severity, duration, plant species, and race of the infecting fungus. Under these circumstances, DNA oxidation and methylation are highly likely to occur. Single- or double-strand breaks can be observed, and most of the time, the repair mechanism fails; eventually, cell death occurs. Since antioxidants are depleted, crop quality also deteriorates even if the plant survives. To the best of our knowledge, most of the interactions between drought and *Z. tritici* on wheat plants have been the subject of additive or synergistic interactions. Cells mostly divide with damaged DNA; newly formed cells do not function properly, ROS cannot be removed, and hormonal homeostasis cannot be maintained with external applications of antioxidants or signalling molecules. Molecular technologies might help to breed multiple stress-tolerant crop plants. Under normal conditions, most of the energy is allocated to quality-related physiological processes, whereas only a limited amount is directed toward defense mechanisms. However, under combined stress conditions, the majority of the energy is redirected toward defense responses, leading to a reduction in quality parameters. Consequently, premature senescence and sudden death syndrome become inevitable outcomes. Combined stress often leads to excess ROS accumulation, membrane instability, and higher MDA levels. Accumulation of shock proteins following drought stress may play significant roles in disease resistance, whereas accumulation of PR proteins following *Z. tritici* infection may play significant roles in drought tolerance, depending on the wheat plant species and stress severity. The important issue to be elucidated here is: “Can wheat plants under both stress factors accumulate those metabolites to delay the onset of stress symptoms?” Since plants under combined stress cannot freely take up water and transport it upward due to stomatal closure, this affects photosynthetic capacity and disrupts the balance between anabolism and catabolism, favouring catabolism. Therefore, impairment of the metabolism of the plant is inevitable. Only tolerant and resistant wheat plants can cope with the negative effects of combined stress. We expect that wheat plants should at least cope efficiently with one of the stress factors. Molecular technologies and nanotechnological approaches hold promise for advancing progress under these stress conditions. When preparing this figure, responses of metabolites from a resistant or tolerant plant were taken into account.

Arrows indicate induction and bars indicate inhibition. Sig: signaling; ST: signal transduction; TFs: Transcription factors; ROS, reactive oxygen species; ABA, abscisic acid; GA, gibberellic acid; SA, salicylic acid; JA, jasmonic acid; ET, ethylene; LPO, lipid peroxidation. P: proline; GB: glycine betaine; T: trehalose; R: raffinose; M: mannitol; S: sorbitol; ssB: single-strand breaks; Phe: phenolic compounds; Aen: antioxidant enzymes; Amol: antioxidant molecules; ABA: abscisic acid; GA: gibberellic acid; SA: salicylic acid; JA: jasmonic acid; ET: ethylene; G: glutathione; C: careotenoids; Glu: glucose; OA: organic acid; A: arginine. The direction of arrows shows “up” and “down” regulation of pathways or metabolites. Green colour shows improvement, and yellow colour indicates stress.

Although we benefited from almost all references cited in this review, we apologize for those whose works were not cited due to time and space limitations. We aimed to reach every point to elucidate drought, *Z. tritici*, and their combined effects. Metabolic responses have been symbolically adopted from the works of [2,88].

In general, pathogens trigger the production of ROS and reactive nitrogen species (RNS), causing oxidative stress and leading to physiological, biochemical, and molecular modifications in cells [30]. Abiotic stressors such as salinity, drought, heat, cold, and environmental pollution also induce the production of ROS and RNS in cells. If a pathogen and an abiotic stress factor occur simultaneously or sequentially in a plant ecosystem, severe stress is inevitable, even if the crop plant is genetically engineered for resistance or tolerance to one stress group. Under these circumstances, plants tend to increase their antioxidant levels to cope with the negative effects of ROS and RNS as best they can. Crop plants, in general, tend to follow a different pattern under stress conditions, e.g., they produce fewer seeds or fruits to survive. However, if they are exposed to multiple stressors, the production of seeds or fruits is significantly reduced, and the quality deteriorates [129]. If a crop plant is moderately resistant or tolerant to either biotic or abiotic stressors, it may survive and produce seeds or fruits, depending on the severity or duration of the combined stressors.

**Figure 7:**
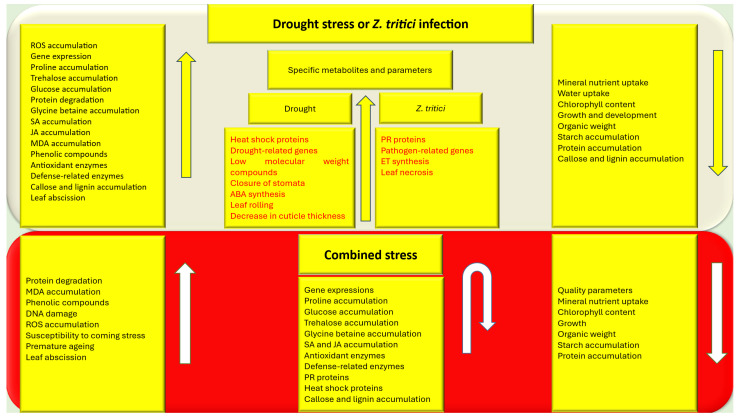
Metabolites that are used to assess the stress factors in drought, *Z. tritici* infection or both.

Although drought is expressed in plant roots, its effects are observed mainly in the photosynthetic parts. When leaf pathogens are involved, we could expect pathogenic effects to be minimized due to water stress. But the increased relative humidity in the early mornings would encourage sporulation, dissemination, and even penetration of the fungus. If we consider that *Z. tritici* could tolerate drought stress compared to wheat plants, the effect of pathogen and drought would be more pronounced than that of either stress factor alone [81]. One of the recent studies showed that *Z. tritici* could be mobilized and stimulated in wheat leaves grown in saline-irrigated soils [12]. The pathogen synthesised relatively higher concentrations of extracellular proteins, proteases, and laccases. *Z. tritici*, a semi-biotrophic pathogen, cannot extend its mycelia for no reason; it can only show increased growth when it is stimulated under drought stress. Our results showed that (unpublished) the pathogen could tolerate drought better than wheat, and recent studies supported this view [42]. One of the main reasons behind this is that the relative humidity in the air, especially early in the morning, could ease the dissemination and penetration of the pathogen [130]. Metabolites that will be used to assess drought stress, *Z. tritici* infection, or the combined stress factors are briefly illustrated with their up- and down-regulation pathways in Figure 7.

Physiological, biochemical, and molecular parameters have been changing under drought stress, *Z. tritici* infection, or both. Upward arrows show ascending, while downward arrows show decreased levels of metabolites or pathways. The U-turn arrow indicates that stress metabolites and parameters increase significantly in the first stage, then decline rapidly as active metabolites diminish.

When wheat is exposed to drought and *Z. tritici* infection simultaneously or sequentially, several processes can lead to DNA damage and reduced genome integrity in plant cells. Although few studies on temperature and drought stress have assessed DNA damage in plants, it is evident that temperature or drought directly results in the formation of various forms of DNA damage [131]. These effects arise mainly from oxidative stress, toxin production, and impaired cellular systems. Although oxidative stress damages all cellular components, it can lead to DNA oxidation and damage even when it persists chronically. If pathogenic stress is involved, additive negative effects on DNA molecules are inevitable. Genomic instability and DNA strand breaks could be observed [132]. We know that drought and other abiotic stresses can lead to genomic instability; the combination of drought stress and *Z. tritici* infection significantly impairs cellular metabolism. However, little is known about their combined effects on DNA damage or strand breaks. However, this gap should be elucidated in detail, and the mechanism should be revealed. Upon the revelation of the DNA damage mechanism, we could study the repair mechanism and the compounds which would help repair DNA molecules. We are aware that rewatering after drought might allow tolerant plants to recover in the same way as plants recovering from pathogen attack; however, combined stress, whether sequential or simultaneous, has distinct effects and needs to be studied in detail with respect to DNA integrity and damage. Since damaged DNA can undergo various stages, such as recovery or programmed cell death (PCD), we could encourage PCD to increase recovery capacity and prevent cell division in cells with damaged DNA by modulating defence pathways. Li et al. [133] stated that drought stress reduced cell viability, impaired cell structure, accelerated nuclear deformation, and increased mitochondrial dissolution, resulting in genomic instability and reduction in DNA integrity of winter wheat cultivars. Nucleic acid hydrolase activity was remarkably high, and nucleic acid concentrations were low. The authors also stated that drought stress significantly increased the expression of four ABA-related genes (*nced1*, *nced2*, *ao1*, *ao2*) while downregulating the expression of four ethylene-related genes (*ers1*, *ers2*, *etr1*, *etr2*). Programmed cell death at earlier stages of drought in the endosperm of winter wheat was also evident. DNA integrity can be elucidated in detail using small RNAs (microRNAs and small interfering RNAs). Their roles in regulating gene expression will enable researchers to explore gene functions and their associated metabolites.

## 7. Mitigation Strategies in Wheat Plants Under the Combined Stress Conditions

**Figure 8:**
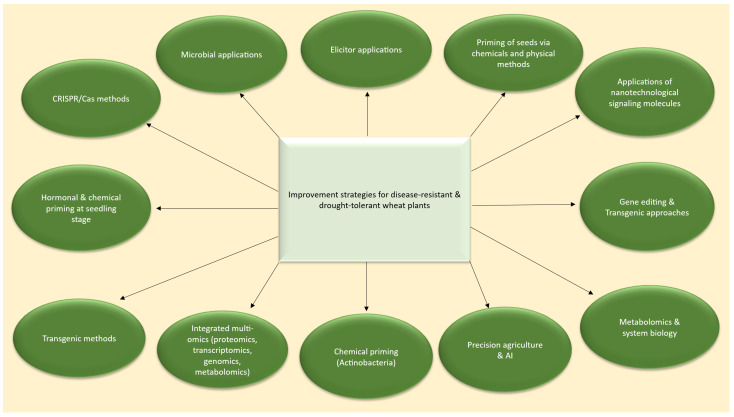
Mitigation, breeding, and remediation strategies for wheat plants grown under drought, pathogen, and combined stress conditions [32,33,109,134,135,136,137].

As abiotic and biotic stress factors become more complex and interact, mitigation strategies gain greater importance. However, these strategies are becoming harder to establish, as the combination of stresses is becoming more complex than previously thought. To breed a disease-resistant crop plant, we may transfer a disease-resistance gene into the target plant to increase its resistance. However, this may not be enough if an additional stress factor is involved. We should employ a multigene resistance program to breed wheat plants for combined stress conditions. We could progress towards ‘Clustered Regularly Interspaced Short Palindromic Repeats/CRISPR-based (CRISPR/Cas)’ systems for the generation of wheat plants. We could identify specific regions of DNA and metabolites and proceed accordingly. However, any suggestions, including biochemical, molecular, and nanotechnological approaches, should be evaluated in detail to avoid unforeseen susceptibility in target plants. It is important to mitigate at least one stress factor when cultivating wheat plants under combined stress conditions. We could at least address the abiotic stressors to improve conditions for plants under combined stress. Whatever approaches are proposed or practised for the remediation of cereals under combined stress conditions, we cannot sacrifice quality parameters, and our approach should be environmentally friendly, e.g., by using biocontrol agents under extreme conditions, applying signalling molecules, or using nanotechnological compounds to improve immunity in host plants. We summarized mitigation and breeding strategies in Figure 8.

## 8. Future Studies and Conclusions

Stressful conditions in plants can either enhance pathogen virulence or render it ineffective [30]. Under drought conditions, pathogen sporulation and mycelial growth may decrease; however, weakened plant defence mechanisms would be reflected in severe pathogenicity under combined stress. Even disease-resistant plants might be severely affected under the combined stress. Even if defence responses are triggered, crop quality may not be maintained. Therefore, understanding the biochemical behaviour of a pathogen under stress conditions can contribute to the development of more tolerant or resistant crop plants for sustainable agriculture. As our understanding of drought and pathogen stress concepts and their mode of action increases, we could generate and breed more tolerant and resistant crop plants without compromising quality parameters. With this approach, the new wheat lines could be produced at a low cost in a short time. Mapping metabolic and genomic pathways would reveal key points for generating new lines. Considering minor rather than major gene expression could be more beneficial for increasing tolerance or resistance of crop plants without interfering with quality parameters. New signaling molecules triggering defence systems and enhancing nutrient absorption should be sought and used. Next-generation nanotechnology-based compounds and chemicals might enhance fertilizer efficacy and improve nutrient efficiency. The increasing demand for food, deteriorating environmental conditions, and the emergence of newly stress-adapted pathogens have necessitated the development of crops that exhibit tolerance to abiotic stress and resistance to biotic stress. The combined effects of two or more concurrent environmental stresses, or of abiotic and biotic stresses, can be far more detrimental to plants than a single stress, leading to severe agricultural losses. It is very difficult to determine whether a reduction in disease resistance is due to changing environmental conditions or to the pathogen’s aggressiveness. Therefore, we need to develop crop plants with enhanced stress tolerance due to increased population growth and rising global temperatures, which will eventually lead to drought. Under these circumstances, the effects of pathogens have been demonstrated in many studies. Combined abiotic and biotic stresses modify plant defence signaling, which may lead to the activation or suppression of defence responses. Although most mechanisms have considered the devastating effects of combined stress, certain abiotic stresses reduce the severity of pathogen infection in plants. In contrast, certain pathogens enhance the tolerance of some plants to specific abiotic stresses. This could be interpreted as a kind of priming before the actual stress arises. Many authors suggested that enhancing plant resistance or tolerance through antagonistic interactions between abiotic and biotic stressors could reveal defence mechanisms that could benefit growers. Drought stress resulting from climate change will not only affect numerous crops in the future but also strongly influence fungal pathogens. It has been shown that many abiotic stresses influence responses to biotic stresses, and vice versa. As a result of climate change, plants become more susceptible to pathogen attacks, and adaptation of phytopathogens has been shown in numerous studies. One of the main reasons may be the genome size, reproduction rate, and population dynamics, which could enhance genetic variation at a faster rate than in plants and increase the capacity for adaptation to environmental stress. Current agricultural practices are unsustainable, and the problems cited in this review are worsening. Therefore, we need to develop new, effective, and environmentally friendly alternatives to maintain or even increase wheat yields, given that combined stress may replace single stress in the near future. Promising biocontrol agents that survive combined stress conditions can be bioformulated for field use. Also, elucidating the full plant transcriptome in response to single or combined stress factors should be vital for identifying genes related to plant tolerance or resistance. Biotrophic plant pathogens are among the most devastating, continually threatening the production of economically important crops. Their lifestyle is exceptional, as they obtain nutrients from host cells via a haustorium. The haustorium is also essential for the secretion and delivery of effectors into host cells to manipulate the host immune system. *Z. tritici* could be considered as one of the most dangerous pathogens, and it acts as both a necrotrophic and a biotrophic pathogen. Turgay et al. [138] reported that resistance-related genes from Stb-6 and Stb-12 could be used to develop new varieties resistant to *Z. tritici*. Bernasconi et al. [139] reported that *Z. tritici* formed highly diverse fungal populations and colonized the same leaf. They demonstrated that avirulent pathogen strains benefited from virulent strains when mixed, in which virulent strains suppressed the immune systems while avirulent strains colonized the apoplast. The defence mechanism begins here; fungal effectors and plant proteins determine the outcome of disease progression. Wheat proteins interact with fungal small secreted proteins (SSPs). Karki et al. [140] stated that SSPs played significant roles in the successful colonization of wheat cells. These interactions have been observed in other pathogens. For instance, infections by *Z. tritici* predisposed wheat to be colonized by *Pseudomonas syringae* through the suppression of host antimicrobial substances [141]. So, we could conclude that the virulent strains of *Z. tritici* facilitate apoplast colonization of the avirulent strains. As drought-affected areas expand, stress-adapted microbial species will spread across them, and new pathogenic species will emerge, threatening unaffected areas. Since the pathways (ABA-SA/JA) challenge each other, we could speculate that breeding a line of stress-tolerant or disease-resistant plants would not solve the problem of wheat or other crop plants facing combined stress factors. Therefore, we need to take a multifaceted approach to elucidate the challenges involved in breeding wheat plants for cultivation under multiple stress conditions.

## Figures and Tables

**Table 1:** The effects of drought, *Z. tritici*, or drought & *Z. tritici* on wheat plants.

Stress Type	Characteristic Symptoms of Stress Agents on Wheat Plants	References
Drought	Proline (accumulation), ABA (increasing trend), ROS (increasing trend), Photosynthesis (decreasing trend), Stomata (closure), Carbohydrate (decreasing trend), Protein (denaturation)	[16,23,45,121,122,123]
*Z. tritici*	Toxin (fungal metabolites), ROS (overproduction), Lignin (accumulation), Phenolic compounds (increasing trend), Chlorophyll (decreasing trend), Photosynthesis (decreasing trend), Energy (ATP decreasing trend)	[1,55,124,125,126]
Drought *& Z. tritici*	Proline, ABA, Phenolic compounds, and Lignin could show an increasing trend; however, as the duration prolongs, these defence-related compounds show declining trends. Toxins and fungal-related enzymes tend to increase.	[71,88,127,128]

## Data Availability

No new data were created or analyzed in this study.
